# Assessing *Bos taurus* introgression in the UOA *Bos indicus* assembly

**DOI:** 10.1186/s12711-021-00688-1

**Published:** 2021-12-18

**Authors:** Maulana M. Naji, Yuri T. Utsunomiya, Johann Sölkner, Benjamin D. Rosen, Gábor Mészáros

**Affiliations:** 1 University of Natural Resources and Life Sciences (BOKU), Vienna, Austria; 2 AgroPartners Consulting, R. Floriano Peixoto, 120 - Sala 43A - Centro, Araçatuba, SP 16010-220 Brazil; 3 Department of Production and Animal Health, School of Veterinary Medicine, São Paulo State University (Unesp), Araçatuba, São Paulo Brazil; 4 International Atomic Energy Agency (IAEA) Collaborating Centre on Animal Genomics and Bioinformatics, Araçatuba, São Paulo Brazil; 5 Animal Genomics and Improvement Laboratory, USDA, ARS, Beltsville, MD USA

## Abstract

**Background:**

Reference genomes are essential in the analysis of genomic data. As the cost of sequencing decreases, multiple reference genomes are being produced within species to alleviate problems such as low mapping accuracy and reference allele bias in variant calling that can be associated with the alignment of divergent samples to a single reference individual. The latest reference sequence adopted by the scientific community for the analysis of cattle data is ARS_UCD1.2, built from the DNA of a Hereford cow (*Bos taurus taurus—B. taurus*). A complementary genome assembly, UOA_Brahman_1, was recently built to represent the other cattle subspecies (*Bos taurus indicus—B. indicus*) from a Brahman cow haplotype to further support analysis of *B. indicus* data. In this study, we aligned the sequence data of 15 *B. taurus* and *B. indicus* breeds to each of these references.

**Results:**

The alignment of *B. taurus* individuals against UOA_Brahman_1 detected up to five million more single-nucleotide variants (SNVs) compared to that against ARS_UCD1.2. Similarly, the alignment of *B. indicus* individuals against ARS_UCD1.2 resulted in one and a half million more SNVs than that against UOA_Brahman_1. The number of SNVs with nearly fixed alternative alleles also increased in the alignments with cross-subspecies. Interestingly, the alignment of *B. taurus* cattle against UOA_Brahman_1 revealed regions with a smaller than expected number of counts of SNVs with nearly fixed alternative alleles. Since *B. taurus* introgression represents on average 10% of the genome of Brahman cattle, we suggest that these regions comprise taurine DNA as opposed to indicine DNA in the UOA_Brahman_1 reference genome. Principal component and admixture analyses using genotypes inferred from this region support these taurine-introgressed loci. Overall, the flagged taurine segments represent 13.7% of the UOA_Brahman_1 assembly. The genes located within these segments were previously reported to be under positive selection in Brahman cattle, and include functional candidate genes implicated in feed efficiency, development and immunity.

**Conclusions:**

We report a list of taurine segments that are in the UOA_Brahman_1 assembly, which will be useful for the interpretation of interesting genomic features (e.g., signatures of selection, runs of homozygosity, increased mutation rate, etc.) that could appear in future re-sequencing analysis of indicine cattle.

**Supplementary Information:**

The online version contains supplementary material available at 10.1186/s12711-021-00688-1.

## Background

Reference genome assemblies are linear haploid sequences that serve as a standard for the analysis of all genomic data and databases [1, 2]. They provide a framework for variant calling, RNA and other sequencing read alignments, gene annotation, and functional analyses [1]. In common practice, sequencing reads undergo a pipeline that starts with the alignment against the reference genome followed by various downstream analyses depending on the respective aims of the study. In a later stage, the loci of interest obtained from the analyses will be annotated back to the same reference genome to infer the genes that are found at these loci and their functionalities [3–5]. A high level of genetic variation within a species can lead to a lower mapping rate of reads and reference allele bias in variant calling if only a single reference genome is used. Thus, as DNA sequencing costs decrease and novel methodologies emerge, multiple reference genomes are being generated for the same species to attempt to rectify previous drawbacks of the use of single reference genomes [6–8].

The total number of gaps in a genome is one indicator for assessing the quality of de-novo reference assemblies. For example, the current reference human genome version 38 (also known as GRCh38) features around 650 gaps [1, 7]. The latest and most widely used cattle genome release ARS_UCD1.2 has only 386 gaps in its final assembly. This assembly was an update of the UMD3.1 assembly that was based on the same inbred Hereford cow as the DNA source providing 250 × more continuity than its predecessor [9, 10]. In addition, there are two assemblies built from a single F1 cross of a Brahman (*Bos taurus indicus—*referred as *B. indicus* hereafter) dam with an Angus (*Bos taurus taurus—*referred as *B. taurus* onwards) bull. Using parent-specific k-mers, the paternal and maternal haplotypes of the F1 animal were separated, leading to an Angus-specific assembly (UOA_Angus_1) and a Brahman-specific assembly (UOA_Brahman_1). Both assemblies were constructed by combining the latest sequencing technologies of PacBio long-reads, Hi-C data, Bio-nano optical reads and Illumina short reads, respectively resulting in 277 and 302 gaps in the final assemblies, which is less than in GRCh38 and ARS_UCD1.2 [7, 9]. It should be noted that the UOA_Brahman_1 reference sequence is the first published de novo assembly of a *B. indicus* genome.

Although Brahman is considered to be a *B. indicus* breed, its formation has a history of admixture between several *B. indicus* and *B. taurus* breeds [11, 12]. It has been estimated that the current Brahman population retains 10% of *B. taurus* ancestry on average [12]. Part of this taurine ancestry has been retained due to artificial selection for the introgression of productivity and reproduction-related traits from those *B. taurus* breeds into the indicine background [11]. Conversely, the high indicine content in the Brahman genome ensures that this breed exhibits most of the traits that are considered beneficial in hot and humid climates, such as heat tolerance and resistance to parasites and infectious diseases [12, 13].

The main objective of using a *B. indicus* instead of a *B. taurus* assembly to guide alignments of re-sequencing data of indicine cattle is to mitigate bias in variant calling. Since *B. taurus* and *B. indicus* diverged about 300,000 years ago, mutation and drift fostered the accumulation of many base substitutions and structural variations between the two subspecies, which in practice may lower the alignment performance of short sequence reads [14, 15]. Indeed, the percentage of indicine reads that are properly aligned against a taurine reference genome has been found to be slightly lower as compared to taurine re-sequencing data [16, 17]. In addition, the number of variants detected in indicine data tends to surpass that of taurine data by millions of variants, which indicates assembly bias during variant calling [18]. In this study, our aims were to (1) examine possible reference genome bias by comparing the number of variant sites detected when using the ARS_UCD1.2 and UOA_Brahman_1 reference sequences; (2) identify genomic regions containing traces of *B. taurus* introgression into the UOA_Brahman_1 assembly; and (3) explore the genes within these regions of interest that may have been selectively retained.

## Methods

### Datasets

We used datasets from NCBI retrieved in FASTQ format from the mirror server at ebi.ac.uk. The dataset consists of *B. taurus* and *B. indicus* cattle from several breeds as listed in Table 1; details for each individual are provided in Additional file 1: Table S1. In total, we used whole-genome sequence data on 112 individuals (54 *B. taurus* and 58 *B. indicus*) from 15 breeds (6 *B. taurus* and 9 *B. indicus*).

**Table 1:** List of FASTQ reads dataset

Breeds	N	Read length	Total reads	Cov	ARS_UCD1.2 (%)			UOA_Brahman_1 (%)		
					Map	Filt	Ret	Map	Filt	Ret
Angus^a^	9	151	172	7.26	97.65	13.74	83.91	97.77	14.99	82.77
Hereford^a^	8	99	242	10.17	99.34	15.83	83.51	99.47	17.26	82.21
Holstein^a^	9	100	124	4.58	93.37	16.82	76.55	93.45	17.91	75.54
Jersey^a^	7	100	186	6.86	98.79	19.79	79.00	98.84	20.84	78.00
Shorthorn^a^	5	99	230	8.36	99.06	19.06	80.00	99.13	20.33	78.80
Simmental^a^	16	100	217	8.59	92.91	17.02	75.89	92.98	18.28	74.70
Bohai^bc^	3	150	222	12.21	99.55	25.12	74.44	99.68	26.40	73.28
Boran^b^	10	101	297	11.03	99.54	15.49	84.04	99.52	15.94	83.58
Brahman^b^	7	114	283	11.64	88.74	3.98	84.76	89.84	4.58	85.26
Gir^b^	9	99	91	3.27	97.09	12.40	84.69	95.64	12.32	83.33
Indian zebu^b^	5	100	152	5.56	98.83	33.05	65.79	98.77	33.12	65.64
Kenana^b^	6	101	307	11.40	99.50	14.59	84.91	99.51	14.68	84.83
Mangshi^b^	7	100	101	3.68	98.29	17.50	80.80	98.25	17.64	80.62
Nelore^b^	6	99	111	4.00	98.18	13.35	84.83	98.05	13.85	84.20
Ogaden^b^	5	101	291	10.80	99.51	15.65	83.86	99.49	16.11	83.38

N: Number of individuals; Read length: read length values were derived from the mode of read length from individuals raw reads from each respective breed; Total reads: total reads in million; Cov: coverage values were estimated from total bases in raw reads and using 2.7 Gb as the genome length; Map: mapped values were percentage of reads aligned to the respective reference genome; Filt: filter values were percentage of reads dropped from the BAM files due to not passing the parameters set during the base recalibration step in the genome analysis tool kit (GATK); Ret: retain values were percentage of reads passing the GATK base recalibration step and kept in the final BAM files for the next step of variants calling for the respective reference genome; All values for total reads, coverage values, mapped values, filter values, and retain values are averages of individuals for each respective breed

^a^*Bos taurus taurus*

^b^*Bos taurus indicus*

^c^Highly admixed with *B. taurus* ancestry

### Alignments and single nucleotide variant (SNV) calling

**Fig. 1:**
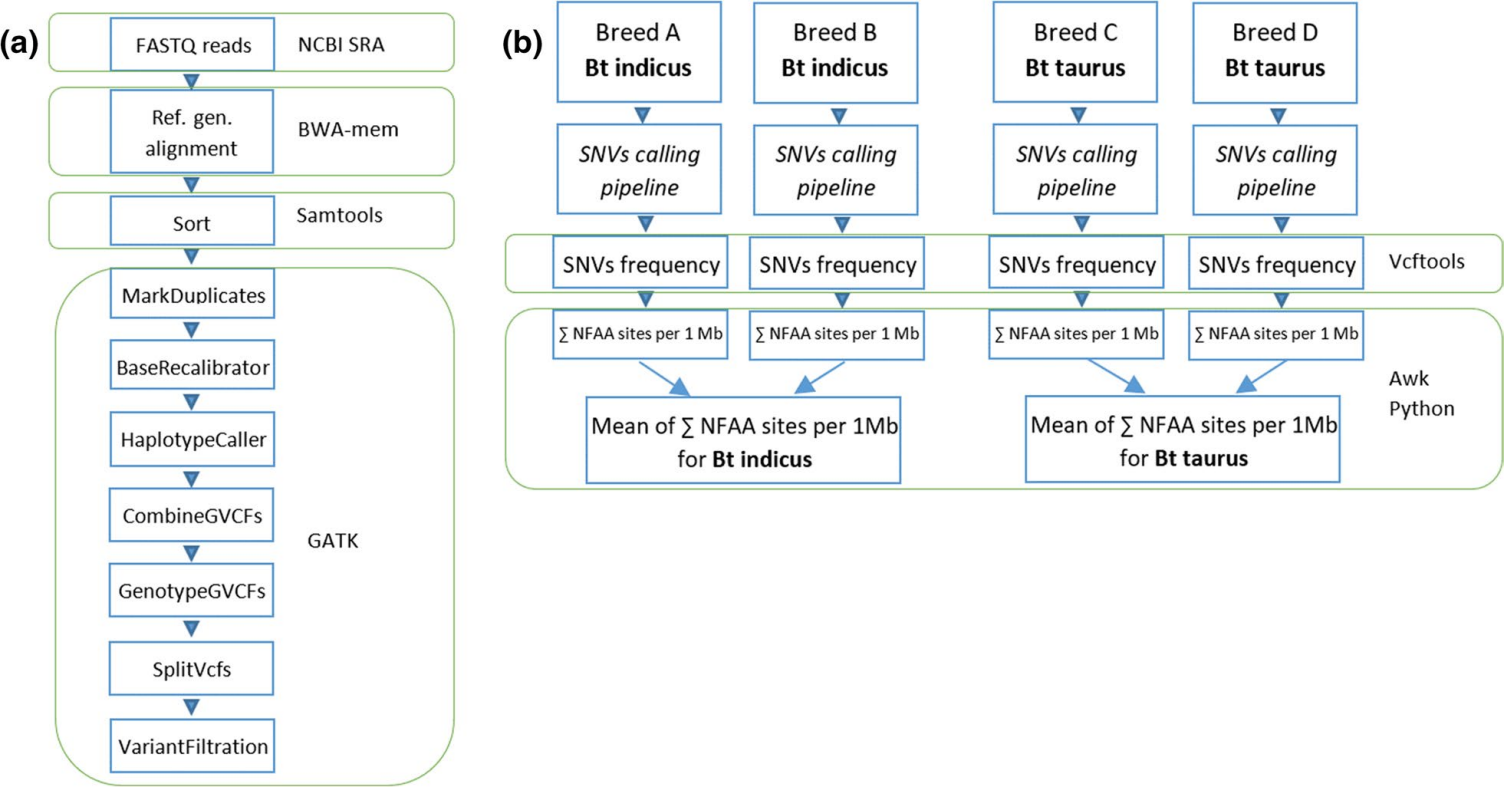
**a** Pipeline for calling single nucleotide variants (SNVs) using fastq reads from the NCBI SRA database and BWA, Samtools, and GATK as the main tools; **b** calculation of the number of NFAA (nearly fixed alternative allele, AA ≥ 0.95) sites for *Bos taurus taurus* and *Bos taurus indicus* sequences aligned to a specific reference genome. We carried out the work flows twice using ARS_UCD1.2 and UOA_Brahman_1 as reference genome, independently

We used the Burrows-Wheeler Aligner (BWA) v.0.7.17 [19], with the maximal exact matches (mem) algorithm and default parameters for mapping paired-end reads to a reference genome. Then, aligned reads in sequence alignment map (SAM) files were sorted by chromosome and consecutively converted to binary alignment map (BAM) files using the sort function of samtools v1.10 [20]. Duplicate reads in each BAM file were flagged using the Picard (https://broadinstitute.github.io/picard/) MarkDuplicates tool embedded in the genome analysis tool kit (GATK v.4.1.0) [21]. Read groups were modified accordingly using Picard AddOrReplaceReadGroups. We applied the BaseRecalibrator and ApplyBQSR functions of GATK v.4.1.0 for base quality score recalibration of each BAM file. We called the genomic variant call format (GVCF) file using the GATK v.4.1.0 HaplotypeCaller with the –ERC GVCF option for each BAM file. We combined the GVCF of samples for each breed using GATK v.4.1.0 CombineGVCFs and subsequently performed GenotypeGVCF with an output of variant call format (VCF) for each respective breed. We performed SplitVcfs to extract SNVs and VariantFiltration to filter variants with the following parameters "QD < 2.0", "QUAL < 30.0", "MQ < 40.0", "SOR > 3.0", "FS > 60.0", "MQRankSum < − 12.5", "ReadPosRankSum < − 8.0". The general pipeline for handling whole-genome sequence (WGS) reads from raw FASTQ up to the variant filtration step, which resulted in a final VCF file for each breed, is shown in Fig. 1a. Unless specifically mentioned, default parameters were used for the tools applied in this study.

A VCF file template is required during the GATK v.4.1.0 BaseRecalibrator step to follow Best Practices as recommended by GATK [5]. This step detected and corrected possible systemic errors in base quality scores of mapped reads, particularly for bases near known variants. For the ARS_UCD1.2 pipeline, we used a VCF file provided by the 1000 bulls genome project [22]. Due to the unavailability of a VCF file template for UOA_Brahman_1 at the time of analysis, we generated an in-house raw VCF file called by bcftools *mpileup* [20] based on a random selection of five Indian zebu and Brahman individuals retrieved from previous studies [11, 23, 24] with accession numbers SRR6423815, SRR4279980, SRR4280002, SRR4280008, and SRR6650038. This template VCF file consisted of ~ 17.5 million variant sites across the genome.

### Hypothesis for the alignment to divergent reference genomes

As indicated by previous studies [7, 18], alignment of individuals against a reference genome built using the same breed results in the detection of fewer SNVs compared to mapping those individuals against a reference genome built from another breed. For instance, consider individual x that belongs to breed A and individual y that belongs to breed B, alignment of individual x against the B reference genome will lead to more detected variants compared to that against the A reference genome and vice versa for individual y. Based on this assumption, we analysed the number of detected SNVs in two scenarios of alignment using ARS_UCD1.2 and UOA_Brahman_1.

### Analysis of SNV frequencies

Pools of FASTQ files from the same breed were processed through the SNV calling pipeline, as shown in Fig. 1b. SNV frequencies were retrieved using vcftools [25] with the –freq2 command, then we applied an ‘awk’ command to retain only the autosomal-diploid sites with a 100% call rate. Then, using an in-house script (nearly fixed alternative allele (NFAA) NFAA_comparisons.py), we retained only the SNVs with a frequency of the alternative allele equal to or higher than 0.95, i.e. SNVs that are almost homozygous for the alternative allele. All codes applied during the analysis are provided in https://github.com/mas-agis/taurus_segment_uoa_brahman.git. For each breed, there were two frequency files, one using the ARS_UCD1.2 and one using the UOA_Brahman_1 assembly as the reference genome. The frequency file is a summary of SNV frequencies with chromosome name, position, reference allele, alternative allele, and the frequencies of reference and alternative alleles within each breed which were obtained as described previously.

As in the Notation 1 shown below, using non-overlapping 1-Mb scanning windows, we sum the number of NFAA sites in both pipelines using the ARS_UCD1.2 and UOA_Brahman_1 assemblies. We plotted ${A}_{i}$ as the sum of NFAA sites across autosomal positions for both reference genome assemblies. Only in the case of the alignment of taurine cattle against the UOA_Brahman_1 assembly, was Notation 2 for window $i$ followed to calculate the delta value ${\Delta }_{i}$ (difference between average number and observed number NFAA sites in each scanning window) by subtracting the mean number of NFAA sites $\mu (A)$ from the total number of NFAA sites ${A}_{i}$ on the respective window $i$. In a single normal distribution, 95% of the data are under $\mu$ ± 1.96 standard deviation (sd), however, the density function of all $\Delta$ values, Additional file 2: Fig. S1, depicts two peaks in the curve instead of a single normal distribution. Therefore, since we were most interested in the second (right) peak which has the largest deviation from the average, we used a threshold of $\mu$ + 1.5 sd. In Notation 3, $T$ denotes the putative taurine-introgressed regions and corresponds to the set of $\Delta$ values that are above our threshold of $\mu$ + 1.5 sd of all $\Delta$ values.

Notation 1: $${A}_{i}= \sum_{i=0}^{i+1000000}{x}_{i}$$;

Notation 2: $${\Delta }_{i}= \mu \left(A\right)- {A}_{i}$$;

Notation 3: $$T=\left\{ \Delta \right| {\Delta }_{i} >\mu ({\Delta }_{i})+1.5\, sd({\Delta }_{i})\}$$.

### Validation

To validate the taurine-introgressed segments in the UOA_Brahman_1 assembly, we carried out principal components (PCA) and admixture analysis using SNVs as inputs. VCF files from the alignment of each breed against UOA_Brahman_1 were converted to plink format. Files from each breed were combined using the merge function of the Plink1.9 software [26]. We set the UOA_Brahman_1 reference genome as an individual that is homozygous for the reference allele at all variant positions (labelled as the Brahman haplotype hereafter) and merged it with the combined dataset. Since the Plink software handles only biallelic sites, we removed 9718 triallelic sites present in the dataset. We inferred the PCA using the plink function of –pca with preset 5 Eigenvalues from 11,135,811 SNVs in the whole genome, 801,366 SNVs in putative taurine introgressed regions and 10,334,445 SNVs in non-introgressed regions of the UOA_Brahman_1 assembly. Eigenvalues were plotted using ggplot2 [27] in R [28].

We used the Admixture v.1.3 software [29] to estimate the population structure of all the individuals and the Brahman haplotype that was inferred from SNVs derived from the whole genome, putative taurine-introgressed, and non-introgressed regions of the alignment against UOA_Brahman_1 as well as SNVs from the alignment of individuals against the ARS_UCD1.2 reference genome. In the Admixture software, we set the number of K to 3, which allowed a clear distinction between *B. taurus* and *B. indicus*. Admixture outputs were plotted using R [28].

### Functional gene annotation

We examined the taurine-introgressed regions in the UOA_Brahman_1 assembly flagged in the previous steps to identify the genes they harbour. Physical genome locations of these segments were re-written to bed format, which includes chromosome number, start and end of the scanning window. The snpEFF software [30] annotated genes from the bed file using the annotation file of the UOA_Brahman_1 version 99 from Ensembl. Genes were assigned to a respective window if their transcripts were located within the window. The genes found within these taurine-introgressed regions were compared to a list of genes that were previously reported to be linked to *B. taurus* genome segments in influential Brahman bulls [11].

## Results

Whole-genome sequence data on 112 individuals from six breeds of *B. taurus* and nine breeds of *B. indicus* ancestry were aligned against the ARS_UCD1.2 and UOA_Brahman_1 reference genomes. In this study, we considered the 29 autosomes with a total length of 2489 Mb for ARS_UCD1.2 and 2478 Mb for UOA_Brahman_1. Table 1 shows the percentages of total aligned reads to each reference genome (Map), percentages of reads removed during the base recalibration process from BAM files due to low mapping rate and duplicate reads in the alignment (Filt). Ret is the total percentage of reads kept in the final BAM files used for SNV calling for the two alignments against the ARS_UCD1.2 and UOA_Brahman_1 reference genomes; details on the individuals included in the study are in Additional file 1: Table S1. A pairwise t-test showed that there was no significant difference (p-value = 0.53) in the total number of aligned reads (Map) between the two alignments of individuals to the reference genomes. However, the test was significant (p-value = 0.0002) when we used the percentage of retained reads (Ret) since the number of reads retained in the final BAM files for all breeds, except Brahman, was smaller for the alignment against UOA_Brahman_1 than that against ARS_UCD1.2.

We called the SNVs for each breed, separately, to detect SNVs that might be unique to one breed. Table 2 shows the number of SNVs in each reference genome using the same quality parameters as for variant filtration. We performed pairwise t-tests for the total number of SNVs from Table 2, separately, for taurine and indicine cattle. A significant difference (p-value = 0.0033) in the total number of SNVs was observed between the alignments of *B. taurus* individuals to each of the reference genomes. Alignment of *B. taurus* individuals against UOA_Brahman_1 resulted in more SNVs than that against ARS_UCD1.2 by an average of 5,015,425 more SNVs.

**Table 2:** Single nucleotide variants (SNVs) for each breed

Breeds	Sub-species^a^	ARS_UCD1.2^b^	UOA_Brahman_1^b^
Angus	*Bos taurus taurus*	11,396,537	17,143,672
Hereford	*Bos taurus taurus*	9,971,428	16,734,834
Holstein	*Bos taurus taurus*	11,133,911	11,459,171
Jersey	*Bos taurus taurus*	9,512,074	15,423,670
Shorthorn	*Bos taurus taurus*	8,464,487	14,393,682
Simmental	*Bos taurus taurus*	14,988,534	20,404,489
Bohai^c^	*Bos taurus indicus*	16,102,113	19,819,636
Boran	*Bos taurus indicus*	27,861,344	27,757,944
Brahman	*Bos taurus indicus*	33,177,201	30,592,708
Gir	*Bos taurus indicus*	24,288,498	22,426,843
Indianzebu	*Bos taurus indicus*	20,210,906	18,518,614
Kenana	*Bos taurus indicus*	26,760,622	24,953,316
Mangshi	*Bos taurus indicus*	21,993,501	20,859,379
Nelore	*Bos taurus indicus*	22,770,177	21,279,805
Ogaden	*Bos taurus indicus*	25,330,919	24,224,387

^a^Sub-species were assigned based on original labels from NCBI-SRA

^b^SNVs passing the variants filtration process respective to the used reference genome

^c^Highly admixed with *B. taurus* ancestry

On the contrary, alignment of *B. indicus* individuals against ARS_UCD1.2 resulted in 895,850 more SNVs than that against UOA_Brahman_1, except for the Bohai breed, for which ~ 3 million more SNVs were found in the alignment against UOA_Brahman_1 than in that against ARS_UCD1.2. Initially, there was no significant difference (p-value = 0.186) in the total number of SNVs in the alignment of *B. indicus* individuals against either reference genome. However, when we re-assigned Bohai as taurine, as the data below suggested was appropriate, we found a significant difference in the number of SNVs (p-value = 0.00069) for *B. indicus* individuals with an average of 1,472,522 more SNVs called when they were aligned against ARS_UCD1.2 than against UOA_Brahman_1. For *B. taurus* individuals, this significance increased (p-value = 0.0011) with an average of 4,830,010 fewer SNVs called when they were aligned against ARS_UCD1.2 than against UOA_Brahman_1.

In the next section, we assessed, separately for each reference genome, how it affected the numbers of NFAA sites instead of the numbers of SNVs in the respective *B. taurus* and *B. indicus* breeds.

### Number of NFAA sites obtained in the alignment against ARS_UCD1.2

**Fig. 2:**
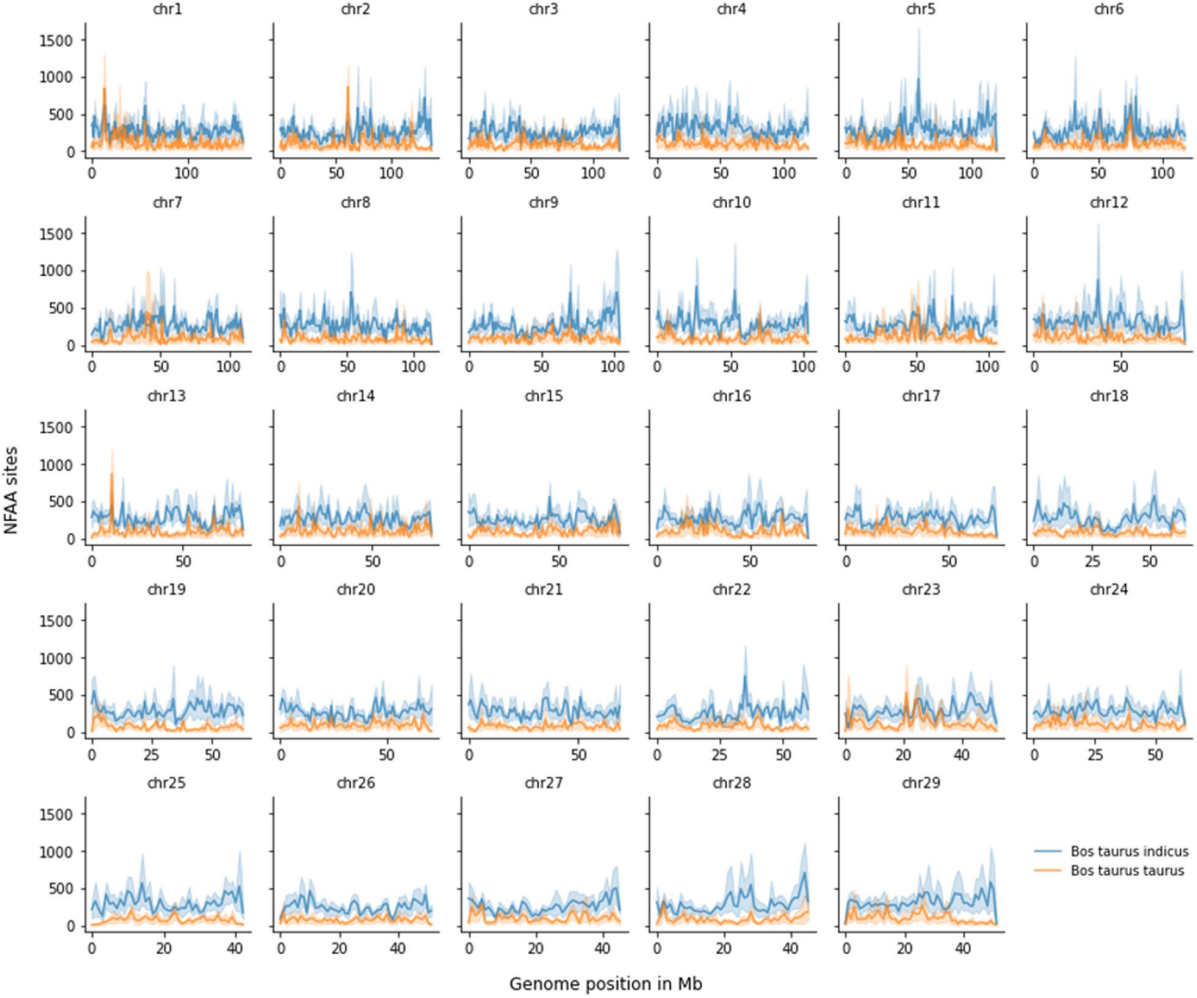
Distribution of the number of SNVs with an alternative allele frequency of 0.95 or higher (i.e., nearly fixed alternative allele-NFAA) using ARS_UCD1.2 as the reference genome (1-Mb scanning windows). Main lines with blue and orange colours are the average numbers of NFAA sites from individuals representing groups of *Bos taurus indicus* and *Bos taurus taurus* and the shadowed blue and orange colors are the actual numbers of NFAA sites for each single *Bos taurus indicus* and *Bos taurus taurus* breed, respectively

The distribution of the number of NFAA sites obtained from the alignments of individuals to the ARS_UCD1.2 assembly is shown in Fig. 2. As expected, the alignment of *B. indicus* sequences against this assembly produced more NFAA sites than that of *B. taurus* sequences, which is consistent with the trend observed in the detection of SNVs. Switching the three Bohai individuals from *B. indicus to B. taurus* did not change this distribution (see Additional file 2: Fig. S2).

An average number of 242 NFAA sites was found in the indicine data across all analysed windows, with a standard deviation of 96 per Mb. The largest number of NFAA sites (901) was on chromosome 5, around the position at 58 Mb, representing approximately one NFAA site per kb. The exceptionally large number of variants in this region extended to the neighbouring chromosomal windows, which presented 366 and 496 NFAA sites. Conversely, we found an average of only 95 ± 68 NFAA sites per Mb in the taurine data. The largest number of NFAA sites was 863, which were located around the position at 11 Mb on chromosome 13. We provide the Z-score transformation of the NFAA sites for alignments to ARS_UCD1.2 in Additional file 2: Fig. S3.

We found a larger number of NFAA sites for indicine than for taurine cattle, regardless of chromosomal position, when reads were aligned to ARS_UCD1.2 (see Additional file 2: Fig. S4). The *B. indicus* group had three times as many NFAA sites than *B. taurus* across all the autosomes: ~ 690,000 sites in *B. indicus* and ~ 238,000 sites in *B. taurus*.

### Number of NFAA sites obtained in the alignment against UOA_Brahman_1

**Fig. 3:**
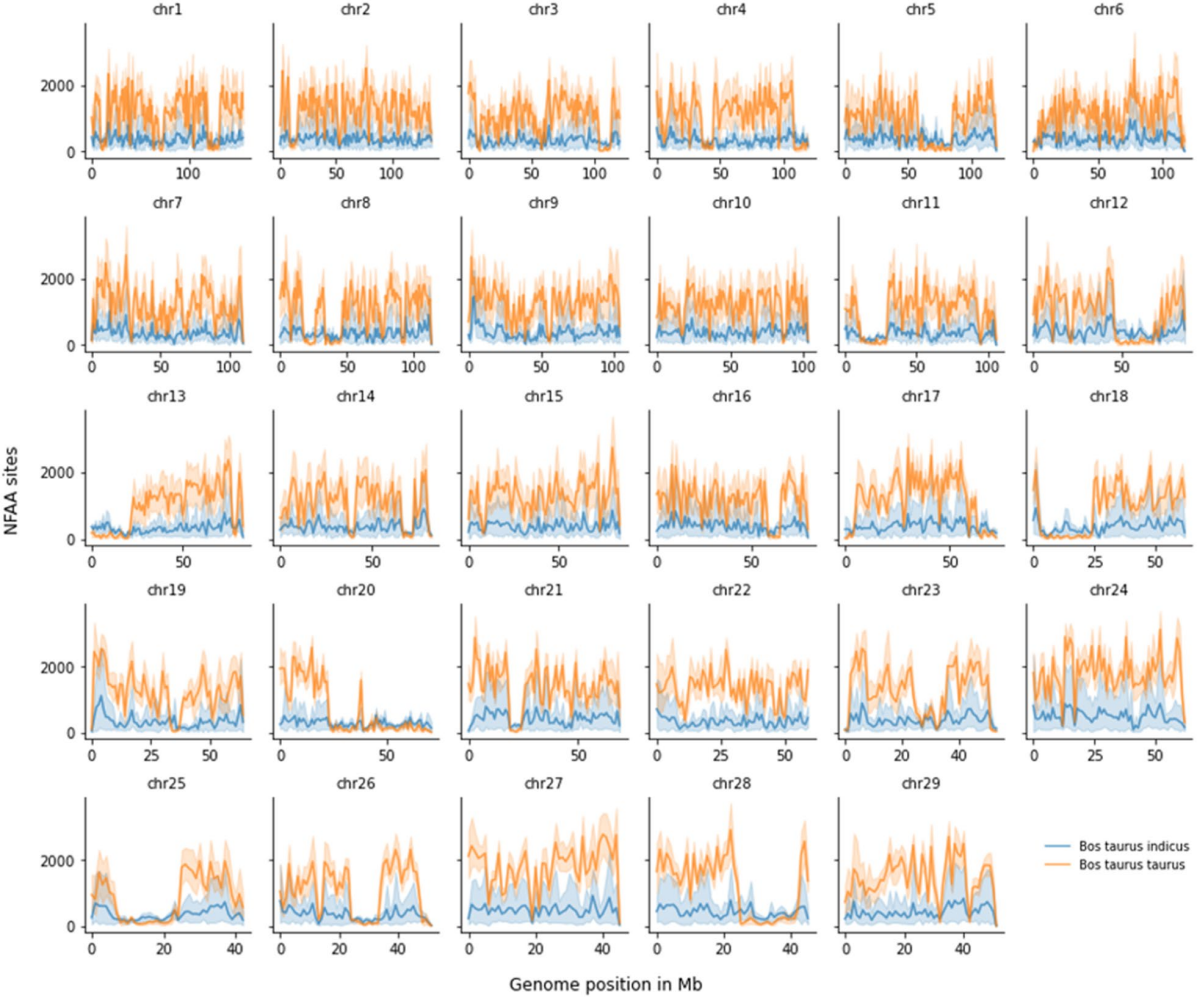
Distribution of the number of SNVs with an alternative allele frequency of 0.95 or higher (i.e., nearly fixed alternative allele-NFAA) using UOA_Brahman_1 as the reference genome (1-Mb scanning windows). Main lines with blue and orange colours are the average numbers of NFAA sites from individuals representing groups of *Bos taurus indicus* and *Bos taurus taurus* and the shadowed blue and orange colors are the actual numbers of NFAA sites for each single *Bos taurus indicus* and *Bos taurus taurus* breed, respectively

The distribution of the number of NFAA sites based on the UOA_Brahman_1 assembly is shown in Fig. 3. Similar to the results in the previous section, for the more distantly related taurine individuals, the number of NFAA sites (average 1162 ± 633 per Mb) was larger than for the indicine individuals (average 345 ± 163 per Mb). Again, classifying the three Bohai individuals as *B. taurus* instead of *B. indicus* did not change this distribution (see Additional file 2: Fig. S5). For *B. taurus*, the window at 52 Mb on chromosome 24 had the largest number of NFAA sites, with a count of 3105 (~ 3 NFAA sites per kb). Additional file 2: Fig. S6 provides a comparison of the boxplot distributions of NFAA sites between *B. taurus* and *B. indicus.*

In addition to the expected results above, we found unique patterns in the alignment against UOA_Brahman_1, which we did not observe in the alignment against ARS_UCD1.2 and which included windows with a small number of NFAA sites for the taurine individuals. Based on our premise that the alignment of less divergent sequences produces smaller numbers of NFAA sites, we conclude that these regions of reduced NFAA counts for *B. taurus* individuals in the alignment against UOA_Brahman_1 identify traces of taurine-introgression in this assembly. These patterns were confirmed in the Z-score transformation of the raw values of NFAA sites (see Additional file 2: Fig. S7).

### *B. taurus* introgression in the UOA_Brahman_1 assembly

**Fig. 4:**
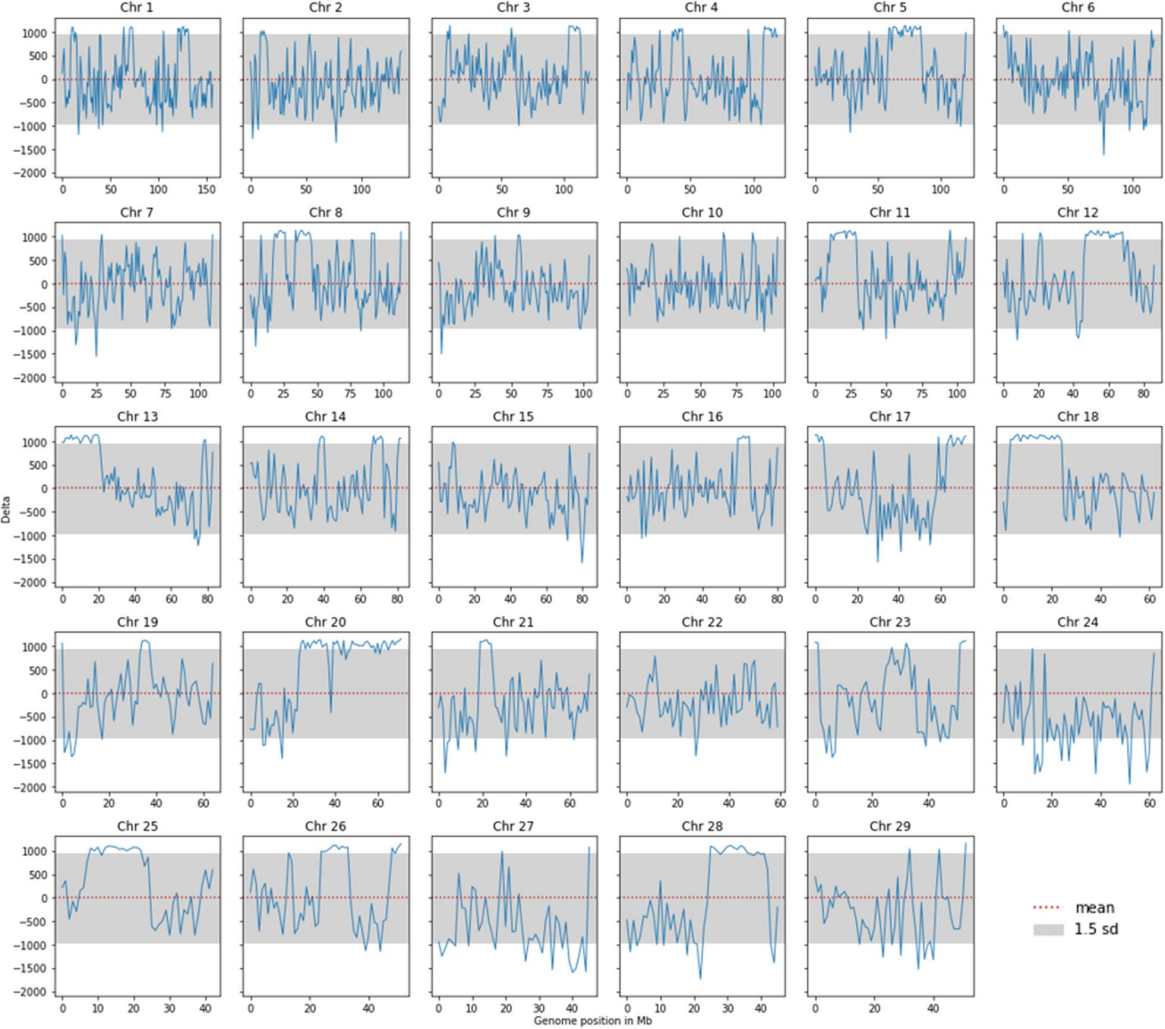
Delta values ($\Delta )$ calculated as the subtraction of the mean number of nearly fixed alternative alleles (NFAA) sites across the chromosomes from the actual number of NFAA sites in each respective scanning window of *Bos taurus taurus* individuals. Taurine-introgressed regions in the UOA_Brahman_1 assembly defined as regions with $\Delta$ values above the arbitrary threshold of 1.5 sd from the mean

We extracted the suspected taurine-introgressed regions in the UOA_Brahman_1 assembly by applying Notation 2 and Notation 3 consecutively, as described in the Methods section. Briefly, the $\Delta$ value is a measure of the difference in the number of NFAA sites in a window and the average number across all autosomes. Values of $\Delta$ are visualized in Fig. 4. Regions with $\Delta$ values greater than 1.5 sd from the mean were considered taurine introgressed regions. These putatively taurine-introgressed regions were found on all the autosomes except chromosome 22 and represented 343 of the 2493 one-Mb windows, which corresponds to ~ 13.7% of the whole genome. By compacting continuous putatively taurine-introgressed regions, this number decreased to 100 segments (see Additional file 3). Forty-seven of these 100 segments were short, i.e. one Mb, and the two longest segments spanned 22 Mb on chromosomes 12 (between positions 47 and 69 Mb) and 18 (between positions 3 and 25 Mb).

### PCA and admixture analyses

**Fig. 5:**
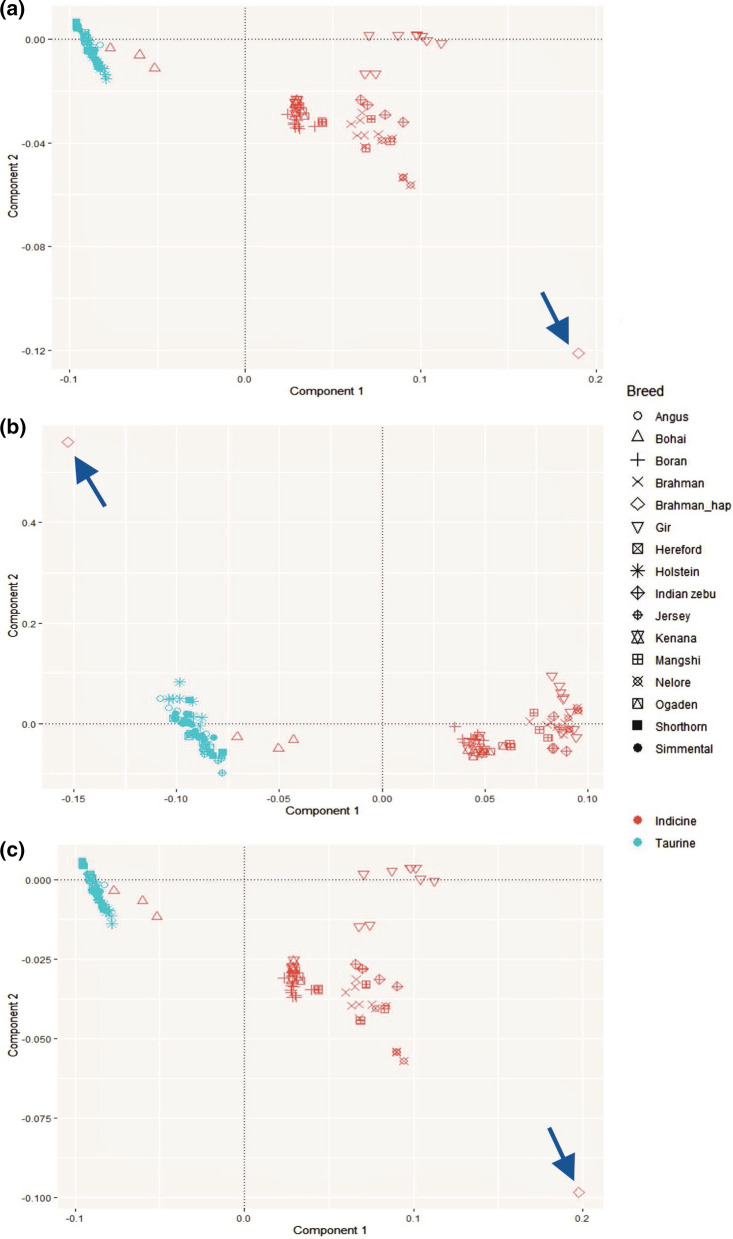
PCA using SNVs from **a** the whole genome; **b** putative taurine-introgressed regions; **c** non-introgressed regions in the UOA_Brahman_1 assembly. Taurine/indicine were assigned based on original labels from NCBI-SRA. Blue arrows point to the Brahman haplotype (SNVs were set to be all homozygous to the reference alleles of the UOA_Brahman_1 assembly)

We used PCA and admixture to validate the position of the Brahman haplotype relative to the genotypes of the other individuals. Figure 5a–c shows the clustering of *B. taurus* and *B. indicus* individuals using SNVs that were derived from the whole genome, the putative-introgressed regions, and the non-introgressed regions in UOA_Brahman_1. The variance explained by principal components 1 and 2 were 72% and 9%, 30% and 8%, and 75% and 10% for each respective PCA. Blue arrows on Fig. 5 show the position of the Brahman haplotype relative to that of the other individuals. In Fig. 5a and c, which is based on SNVs from the whole genome and non-introgressed regions, the Brahman haplotype is positioned in the lower right of the graph, closest to the *B. indicus* individuals. Conversely, in Fig. 5b, which is based only on SNVs from genome regions flagged as taurine-introgressed, the Brahman haplotype is positioned in the upper left of the graph, closest to the *B. taurus* cluster. It should be noted that the Bohai breed clusters with the *B. taurus* individuals in all three plots.

**Fig. 6:**
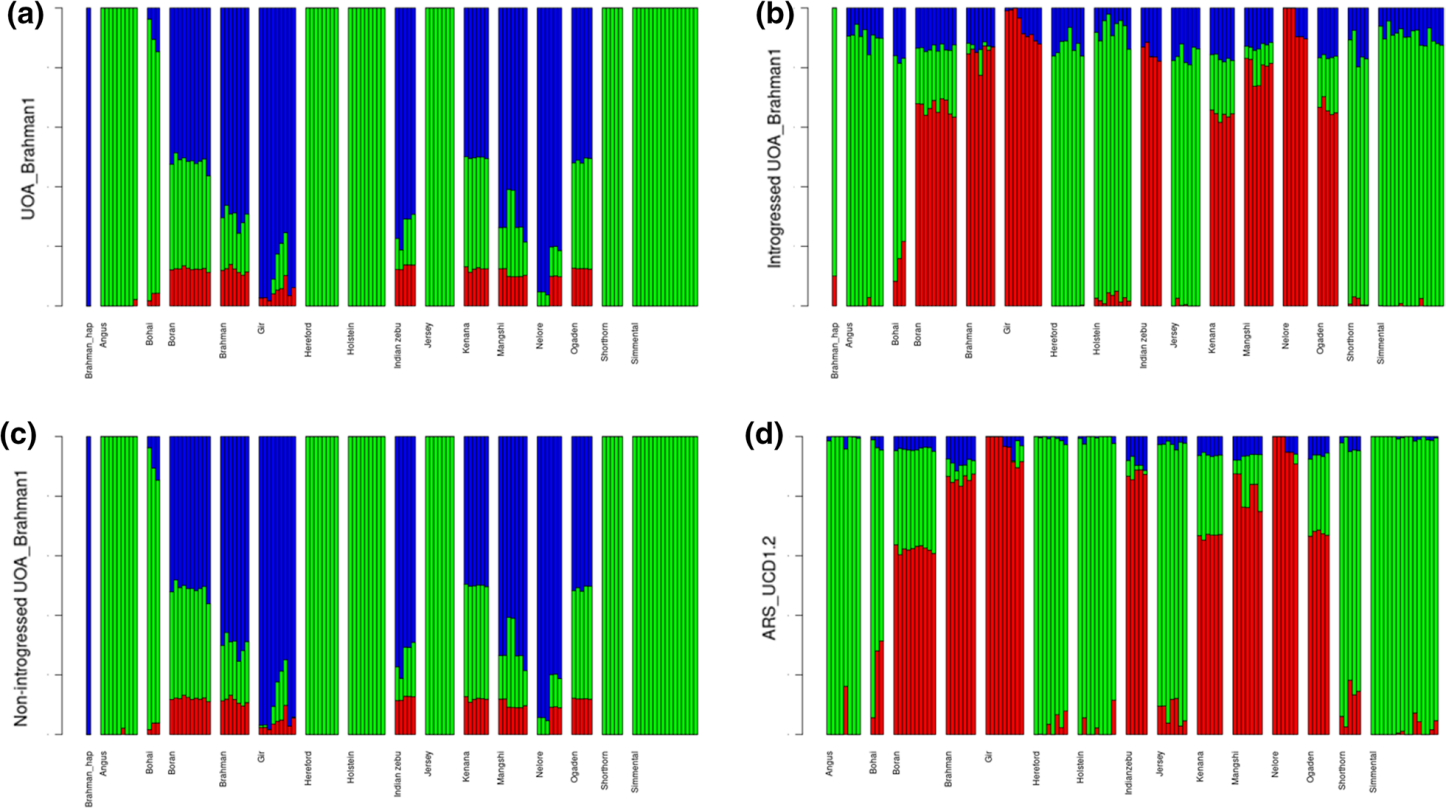
Admixture analysis using SNVs from **a** the whole genome; **b** putative taurine-introgressed; **c** non-introgressed regions in the UOA_Brahman_1 assembly. Brahman_hap is an artificial individual with all the genotypes homozygous to the reference alleles of the UOA_Brahman_1 reference genome. **d** Admixture inferred using SNVs derived from the alignment against the ARS_UCD1.2 assembly. Green, red and blue colors depict subpopulations K1, K2, and K3, respectively

The results of the admixture analysis using the SNVs from the whole genome (~ 11 million SNVs), the putative taurine-introgressed (~ 801,000 SNVs), and the non-introgressed regions (~ 10 million SNVs) in the UOA_Brahman_1 assembly are in Fig. 6a–c, respectively. Figure 6a and c show that the Brahman haplotype is again positioned closest to the *B. indicus* individuals, with an admixture pattern that is very similar to that of the *B. indicus* Boran, Brahman, Gir, Indian zebu, Kenana, Mangshi, Nelore and Ogaden breeds, while Fig. 6b shows that it has a population structure similar to that of the *B. taurus* Angus, Hereford, Holstein, Jersey, Shorthorn and Simmental breeds. In addition, these admixture analyses suggests that there are still fractions of the *B. indicus* genome, up to a tenth, in the flagged introgressed-regions of the UOA_Brahman_1 reference genome. Figure 6d, which is derived from SNVs (~ 17 million SNVs) obtained in the alignment against ARS_UCD1.2, shows similar patterns of K distributions among the breeds as that inferred from the taurine-introgressed regions in Fig. 6b. Again, the Bohai breed appears to be more similar to *B. taurus* than *B. indicus.* In the Admixture software, we chose K = 3 for the plots in Fig. 6 because it resulted in the lowest cross-validation errors, i.e. 0.632, 0.746, 0.621, and 0.652 for all the SNVs from the whole genome, from the putative taurine-introgressed and the non-introgressed regions obtained in the alignment against UOA_Brahman_1, and from the whole genome obtained in the alignment against ARS_UCD1.2, respectively (see Additional file 2: Fig. S8).

### Annotation of taurine-introgressed regions

We annotated the putative taurine-introgressed regions in the UOA_Brahman_1 assembly and found 2226 genes. The genes for each taurine segment are listed in Additional file 3. Sixty-six of the genes in 19 segments were previously reported to be under positive selection in 40 influential Brahman bulls [11] as shown in Table 3.

**Table 3:** *B. taurus* introgressed segments in the UOA_Brahman_1 reference genome that overlap with positively selected genes in the Brahman population as reported by [11]

Chr	Start (Mb)	End (Mb)	Total $\Delta$ ^a^	Size (Mb)	Positive genes
3	58	59	1090.88	1	*BCL10, C1orf52*
5	43	44	1020.05	1	*SYT10*
6	2	4	2016.60	2	*ANXA5, BBS7, EXOSC9*
7	0	1	1034.72	1	*STARD4*
7	29	30	1052.05	1	*ACOT12, ANKRD34B, CKMT2, FAM151B, RASGRF2, SSBP2*
10	86	87	1094.05	1	*CEP128*
11	11	13	2084.10	2	*CYP26B1, DYSF, EMX1, EXOC6B, SPR*
11	14	30	17237.41	16	*BIRC6, CRIM1, DPY30, MEMO1, NLRC4, SLC30A6, SPAST, TTC27, YIPF4*
11	95	96	1144.55	1	*MAPKAP1, PBX3*
12	11	12	1076.72	1	*NAA16, VWA8*
13	0	21	22459.58	21	*AHCY, DYNLRB1, MAFB, MAP1LC3A, PIGU, TOP1*
14	67	68	1117.55	1	*SDC2*
14	81	83	2138.93	2	*SNTB1*
16	59	66	7608.05	7	*ZNF648*
17	0	5	5367.15	5	*TTC28*
23	32	33	1068.35	1	*ABT1, HFE, PRSS16, SLC17A2, TRIM38, ZNF184, ZNF391*
25	12	23	11676.41	11	*AQP8, LCMT1, SCNN1G, USP31*
26	13	14	964.55	1	*RAB11FIP2*
26	24	34	10574.31	10	*ABCC2, CHUK, COX15, CPN1, CRTAC1, CUTC, DNMBP, ENTPD7, ERLIN1, SFRP5, SORCS1, ZFYVE27*

$\Delta$: subtraction of the mean number of nearly fixed alternative allele (NFAA) sites across each chromosome from the total number of NFAA sites in each respective scanning window of *B. taurus* dataset; Total $\Delta$: sum of $\Delta$ on each continuous putative taurine segment

## Discussion

The reference genomes, ARS_UCD1.2 and UOA_Brahman_1, were built from Hereford-*B. taurus* and Brahman-*B. indicus* haplotypes, respectively. Using high-density single nucleotide polymorphisms (SNPs), O’Brien et al. [31] reported a fixation index (F_st_, i.e. a measure of population differentiation) of 0.374 for Hereford and Brahman populations, which is higher than the within-subspecies F_st_ values of 0.068, 0.160, 0.047, 0.044 for Holstein-Hereford, Angus-Hereford, Gir-Brahman, Nelore-Brahman, respectively [31]. Previous studies [7, 18] reported that mapping individuals to a reference genome that is built using the same breed resulted in the detection of fewer SNVs than mapping them to a reference genome built from another breed, which is consistent with our results. Indeed, the alignment of *B. taurus* individuals against UOA_Brahman_1 resulted in ~ 5 million more SNVs while the alignment of *B. indicus* individuals against ARS_UCD1.2 resulted in only ~ 1.5 million more SNVs. This discrepancy in the number of SNVs detected for cross-subspecies alignments is possibly due to the degree of breed purity. As the results of the admixture analysis show, the *B. taurus* individuals were more uniform while the *B. indicus* individuals displayed a greater level of diversity. It should be noted that the Bohai breed seems to be highly admixed with *B. taurus* ancestry and perhaps should not be labeled as *B. indicus* in the SRA database. The results of the PCA and admixture analysis and the trend of the SNVs detected for this breed followed a pattern similar to those of the *B. taurus* breeds. In addition, while the alignment of most of the taurine breeds against UOA_Brahman_1 resulted in additional millions of SNVs, that of the Holstein breed resulted in only 300,000 more SNVs. This number of additional SNVs obtained in the cross-alignment of Holstein against UOA_Brahman_1 is comparable to that obtained when indicine sequences are aligned against ARS_UCD1.2. Nevertheless, our findings support that cross-subspecies alignment leads to the detection of more SNVs in agreement with previous studies [7, 18].

As the detected SNVs may contain the reference allele, either in the homozygous or heterozygous state, we further explored the effect of cross-subspecies alignments on the number of SNVs with a frequency of alternative alleles higher than 0.95 (NFAA). Based on the results of SNV detection in cross-subspecies alignments, we expect a similar trend for the NFAA sites. To quantify this, we counted the number of NFAA sites for each breed using non-overlapping 1-Mb windows to reflect the one centimorgan (cM) genetic distance [32]. In the alignments against ARS_UCD1.2, we obtained different numbers of NFAA sites within 1-Mb windows between the *B. indicus* and *B. taurus* cattle groups. The indicine group presented nearly threefold more NFAA sites than the taurine group. This could be explained by the fact that ARS_UCD1.2 derives from an inbred Hereford cow that belongs to the *B. taurus* subspecies of cattle. Similarly, in the alignments against UOA_Brahman_1, generally the taurine cattle presented about 3.14-fold more NFAA sites than the indicine cattle. Thus, in addition to an increased number of detected SNVs [7, 18], we confirm that the numbers of NFAA sites also increase in cross-subspecies alignments of individuals.

Interestingly, we detected regions with fewer than expected NFAA sites by mapping *B. taurus* individuals against UOA_Brahman_1, and suggest that these regions are in fact the result of taurine introgression corresponding to approximately ~ 13.7% of the genome. These introgressed regions represent a slightly higher percentage than the reported average of 10% in the Brahman population [7]. Yet, this proportion is still within the range of previously reported estimates among which the maximum taurine proportion found in a single animal was 37% [7]. Studies on Brahman populations reported that *B. taurus* ancestry is present on almost all the chromosomes, with a greater proportion of the segments found on chromosomes 1, 4, 8, and 14 [12] and chromosomes 8, 12, 14, 23, 26 and 29 [11]. Consistent with these results, in the alignment against the UOA_Brahman_1 assembly, we found taurine-introgressed regions on all the chromosomes except 22. It is important to note that our proposed taurine-introgressed regions for this assembly correspond to only one haplotype inherited from the maternal lineage of a single animal. Considering the apparent taurine admixture in the Bohai breed, we re-ran the NFAA analysis by assigning Bohai as taurine. However, since only three Bohai individuals were included in our analysis, this did not alter the definition of the taurine introgressed regions in the UOA_Brahman_1 assembly.

Previous studies have aligned WGS data from individuals of admixed and putative-ancestral breeds against a reference sequence and defined regions of introgression as those containing shared derived alleles [11, 33]. Similarly, in our study, we used alternative alleles as an indicator of introgression. However, the goals of those previous studies were to identify introgressed regions in individuals relative to the same reference genome, while in our study our aim was to detect introgressed regions in the assembly from the perspective of individuals of known pure-breeds. With the ongoing efforts to construct a consensus genome from multiple individuals to represent the broadest possible diversity within a species [1, 6], a similar method of NFAA assessment could be applied to validate that the final consensus genome is not biased.

As supported by the PCA and admixture analyses, the UOA_Brahman_1 haplotype is most similar to that of *B. taurus* individuals in the introgressed regions. The distance of the Brahman haplotype from other samples in the PCA is likely due to sequencing errors in the assembly. The admixture analysis also showed a small fraction of *B. indicus* in the flagged taurine-introgressed regions, which sums up to 10% of the size of the taurine-introgressed regions and could be explained in two ways: (1) the threshold, which was set at the mean plus 1.5 standard deviation of the $\Delta$ values and used to define the taurine-introgressed regions, may be too lax; and (2) our 1-Mb window size could be too large. Thus, this fraction may, in fact, represent false positives in our analysis.

The longest spans of putative taurine-introgressed regions in this assembly were 22 Mb long on chromosomes 12 and 18. Considering all the segments, the average length of introgressed regions is 3.43 Mb. The length of genomic regions with inter-species introgression is strongly related to the history of the breeds since their initial crossing, the breeding scheme, the recombination rates, and the pressure of adaptive/positive selection [11, 12, 34, 35].

We found 2226 genes within putative *B. taurus* regions in the UOA_Brahman_1 assembly. As we flagged 13.7% of UOA_Brahman_1 assembly as taurine-introgressed regions, the number of genes found within these regions was smaller by 823 than the number expected by chance. These 2226 genes were classified into two groups: (1) a group of 66 genes that have been reported to be under positive selection [11], with functions related to productivity and developments traits, as shown in the Results section; and (2) a group of neutral genes that are in randomly segregating putative *B. taurus* regions, since they have not been reported to be under positive selection in Brahman cattle. Although unreported, these genes might be important for the individual itself, i.e. related to productivity and developments traits, which were selected for in the formation of Brahman cattle, such as: *SYT10*, a gene involved in feed efficiency [36]; *BIRC6* the expression of which is considered vital for embryo survival particularly during pre-implantation embryonic development in cattle [37]; *CYP26B1*, which encodes a retinoic acid metabolizing enzyme; it has been shown that *CYP26B1* knock-out impacts the skeletal and limb formation in vertebrate embryos [38, 39]; genes related to binding activities for phospholipid and ATP as indicated by *MAP1LC3A*, *MAPKAP1*, and *SPAST*; and *NLRC4* and *HFE* related to the major histocompatibility complex (MHC) involved in immune responses.

The number of identified SNVs and their allele frequencies in any sample pool are prone to be altered by the individual’s background such as breed type, size, phenotypic performance, relatedness, and also by the software chosen for calling variants [40–44]. Although, we used a relatively small subset of individuals and only a single tool for calling variants (i.e. GATK) to infer the presence of putative *B. taurus* sequences in the UOA_Brahman_1 assembly, we presume that these segments can result in the detection of more SNVs with nearly fixed alternative alleles in the alignments of novel *B. indicus* individuals. This has the potential to falsely identify signatures of selection because the number of alternative alleles, also called derived alleles, is frequently used to identify signatures of selection [45–47].

Choosing one reference assembly over another can lead to trade-offs in downstream genomic analyses. As indicated by our results, aligning individuals against different reference assemblies results in different numbers of detected SNVs and different allele frequencies which are crucial for the accuracy of genome-wide association studies and of analyses of signatures of selection [45–48]. Other points to consider are how well the reference genome builds are supported in various genomic tools and how often is the reference genome used by other studies to enable straightforward comparisons of results [5]. The main reason to switch from ARS_UCD1.2 to UOA_Brahman_1 as the reference genome is to improve sequence read mapping and avoid possible variant calling bias for re-sequencing *B. indicus* individuals. Thus, downstream analysis can be carried out more accurately, since the individual that is used to reconstruct the assembly better represents the breed. Therefore, we highly recommended the use of UOA_Brahman_1 as the reference genome for the analysis of re-sequencing *B. indicus* individuals keeping in mind that it contains taurine segments that need to be accounted for.

## Conclusions

Our results confirm that an increased genetic distance between the sequence data to be aligned and the chosen reference genome results in an increased number of detected variants. We flagged 13.7% of the genome in UOA_Brahman_1 as originating from *Bos taurus taurus*. The genes that are located within these taurine introgressed segments and are reported to be under positive selection in the Brahman population are linked to feed efficiency, reproduction, and immune response traits. These reported taurine-introgressed segments should be taken into consideration in forthcoming re-sequencing analyses of indicine genomes aligned to the UOA_Brahman_1 assembly since they might cause variant calling bias.

## Supplementary Information

**Additional file 1: Table S1.** Accession numbers and alignment details. The table provides the accession numbers of all individuals used as representatives of *B. taurus* and *B. indicus,* as well as details for alignments against both reference genomes (ARS_UCD1.2 and UOA_Brahman_1).

**Additional file 2: Figure S1.** Density function of $\Delta$ values. **Figure S2.** Distribution of nearly fixed alternative allele-NFAA sites considering Bohai as taurine for alignment against ARS_UCD1.2. **Figure S3.** Z-score transformation of nearly fixed alternative allele-NFAA sites distribution for alignment against ARS_UCD1.2. **Figure S4.** Boxplot of nearly fixed alternative allele-NFAA sites for alignment against ARS_UCD1.2. **Figure S5.** Distribution of nearly fixed alternative allele-NFAA sites considering Bohai as taurine for alignment against UOA_Brahman_1. **Figure S6.** Boxplot of nearly fixed alternative allele-NFAA sites for alignment against UOA_Brahman_1. **Figure S7.** Z-score transformation of the nearly fixed alternative allele-NFAA sites distribution for alignment against UOA_Brahman_1. **Figure S8.** Cross-validation error for the admixture analysis.

**Additional file 3.** Putative segments of *B. taurus* introgressed regions in the UOA_Brahman_1 reference genome. The data is in semicolon-separated format. It provides information for the physical location of 100 segments flagged as *B. taurus* introgressed regions in the UOA_Brahman_1 assembly and their delta values and annotated genes. Positive genes are those reported in [11], and neutral genes are found within each respective segment, but are not listed in [11].

## Data Availability

Accession numbers for each individual used in this study are provided in ‘Additional file 1: Table S1’ and can be retrieved from the NCBI repository https://www.ncbi.nlm.nih.gov/sra/. Cattle reference sequences ARS_UCD1.2 (*B. taurus*) are available at the NCBI repository https://www.ncbi.nlm.nih.gov/genome/?term=cattle. Cattle reference sequences UOA_Brahman_1 (*B. indicus*) are available at the NCBI repository https://www.ncbi.nlm.nih.gov/assembly/GCF_003369695.1. Codes and in-house Python scripts are available at https://github.com/mas-agis/taurus_segment_uoa_brahman.git.
