# Supplementary material for: Assessing Bos taurus introgression in the UOA Bos indicus assembly
Source: Genet Sel Evol. 2021 Dec 18;53:96. doi: 10.1186/s12711-021-00688-1 (PMC8684283; doi:10.1186/s12711-021-00688-1)
Supplement: Supplementary file 2 — Additional file 2: Figure S1. Density function of $\Delta$Δ values. Figure S2. Distribution of nearly fixed alternative allele-NFAA sites considering Bohai as taurine for alignment against ARS_UCD1.2. Figure S3. Z-score transformation of nearly fixed alternative allele-NFAA sites distribution for alignment against ARS_UCD1.2. Figure S4. Boxplot of nearly fixed alternative allele-NFAA sites for alignment against ARS_UCD1.2. Figure S5. Distribution of nearly fixed alternative allele-NFAA sites considering Bohai as taurine for alignment against UOA_Brahman_1. Figure S6. Boxplot of nearly fixed alternative allele-NFAA sites for alignment against UOA_Brahman_1. Figure S7. Z-score transformation of the nearly fixed alternative allele-NFAA sites distribution for alignment against UOA_Brahman_1. Figure S8. Cross-validation error for the admixture analysis. [file 12711_2021_688_MOESM2_ESM.pdf]

Additional file 2. Supplementary figures

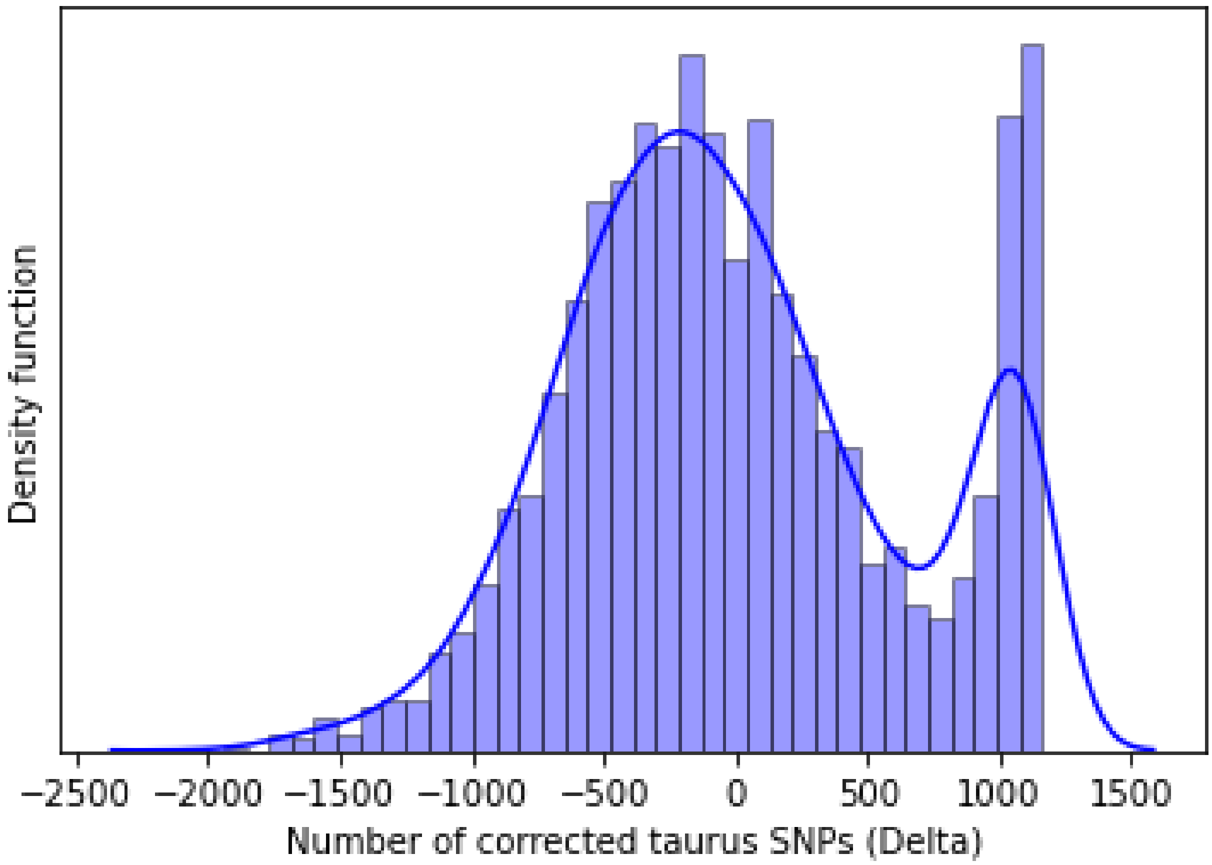

Figure S1. Density function of Δ values. Delta values were calculated as subtraction of mean nearly fixed alternative alleles (NFAA) sites across chromosome from the actual NFAA sites in each respective scanning window of *B. taurus* individuals

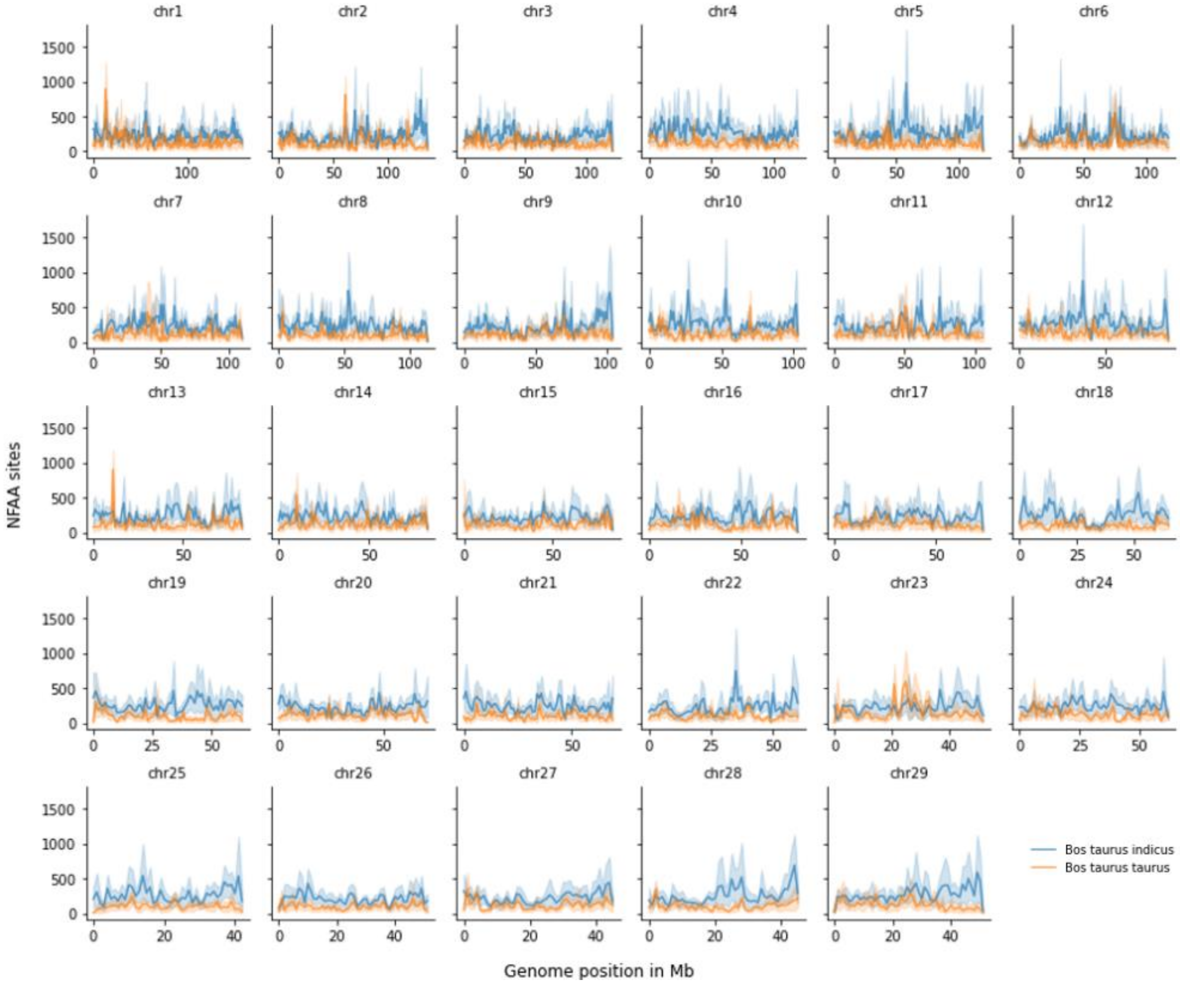

Figure S2. Re-creation of figure 2 from the main text when considering Bohai as taurine (Distribution of the number of SNV with an alternative allele frequency of 0.95 or higher (i.e., nearly fixed alternative allele-NFAA) using ARS_UCD1.2 as the reference genome (1 Mb scanning windows). Main lines with blue and orange colours are the average of NFAA from individuals representing groups of *Bos taurus indicus* and *Bos taurus taurus*. While the shadowed-colors of blue and orange are the actual NFAA for each single breed of *Bos taurus indicus* and *Bos taurus taurus*, respectively)

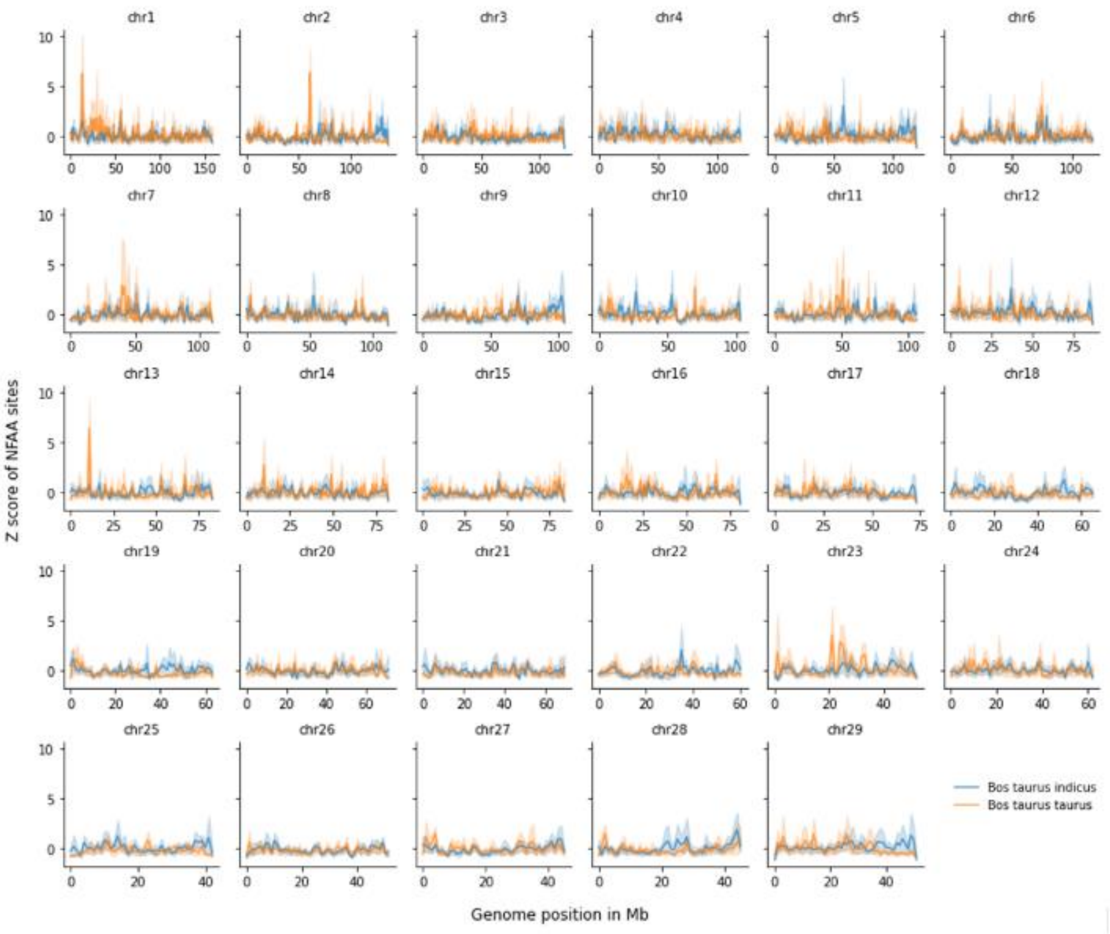

Figure S3. Z-score transformation of the number of SNV with alternative allele frequency of 0.95 or higher (i.e., nearly fixed alternative allele-NFAA) using ARS_UCD1.2 as the reference sequence (1 Mb scanning windows)

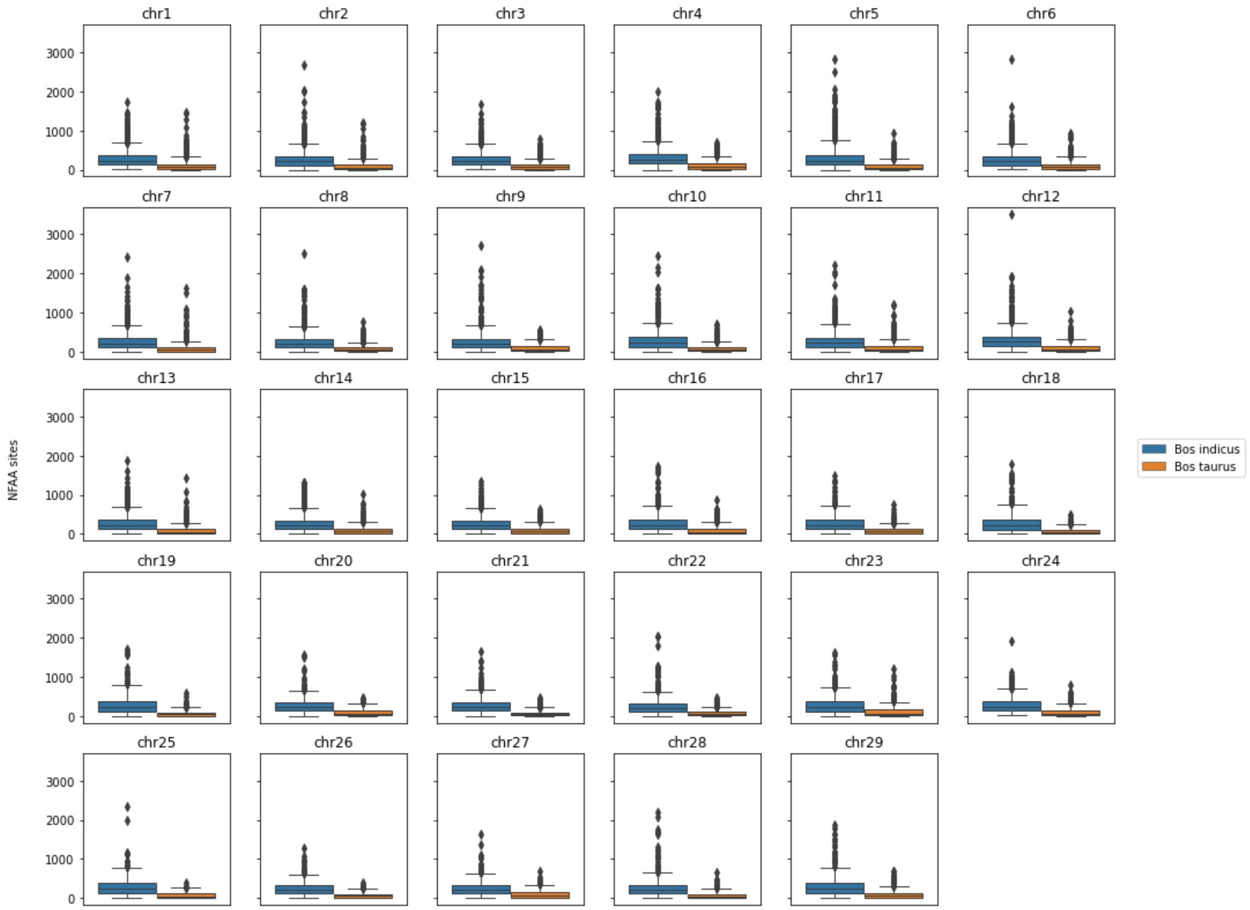

Figure S4. Boxplot of the number of SNV with alternative allele frequency of 0.95 or higher (i.e., nearly fixed alternative allele-NFAA) using ARS_UCD1.2 as the reference sequence (1 Mb scanning windows)

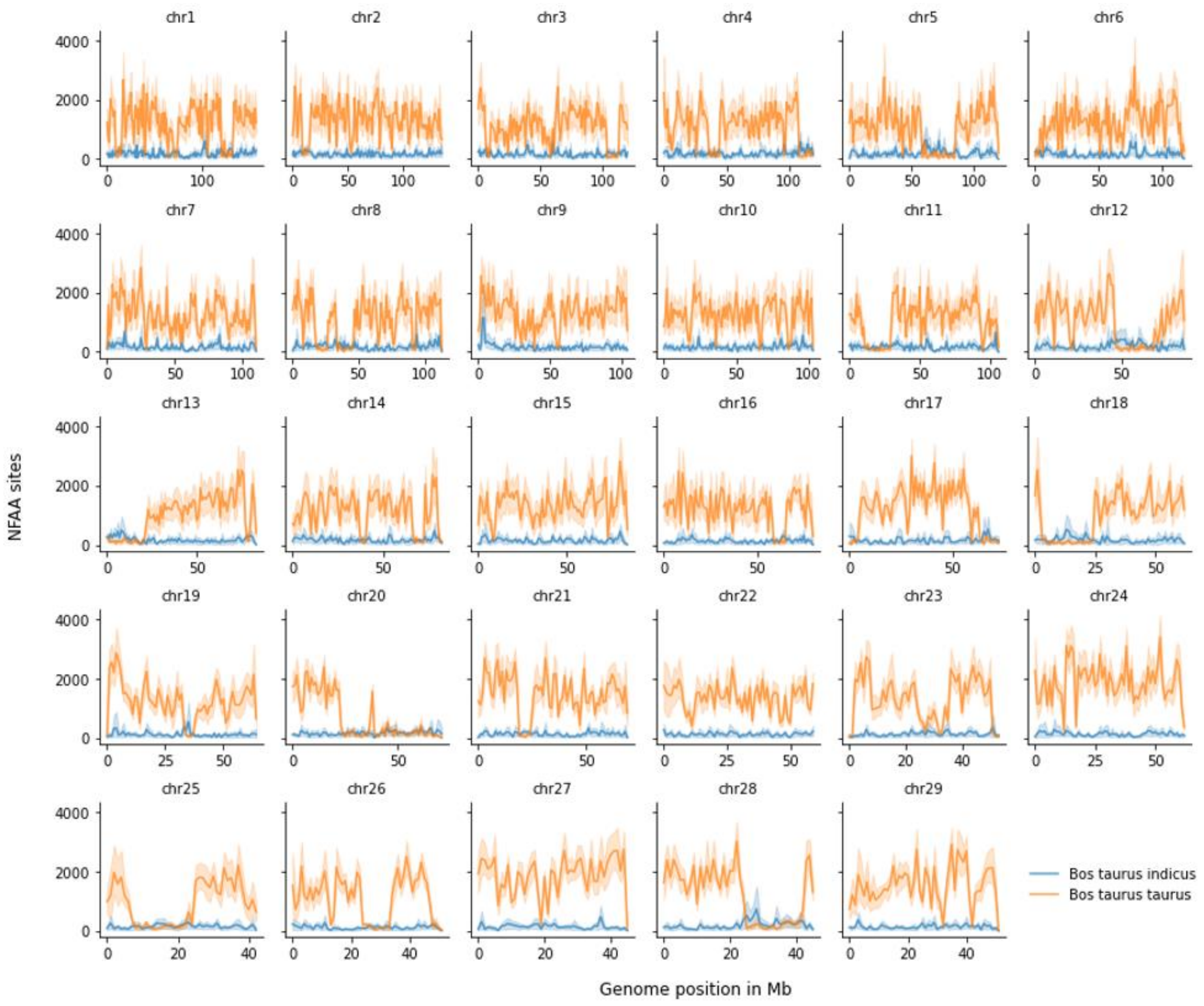

Figure S5. Re-creation of figure 3 from the main text when considering Bohai as taurine (Distribution of the number of SNV with an alternative allele frequency of 0.95 or higher (i.e., nearly fixed alternative allele-NFAA) using UOA_Brahman_1 as the reference genome (1 Mb scanning windows). Main lines with blue and orange colours are the average of NFAA from individuals representing groups of *Bos taurus indicus* and *Bos taurus taurus*. While the shadowed-colors of blue and orange are the actual NFAA for each single breed of *Bos taurus indicus* and *Bos taurus taurus*, respectively)

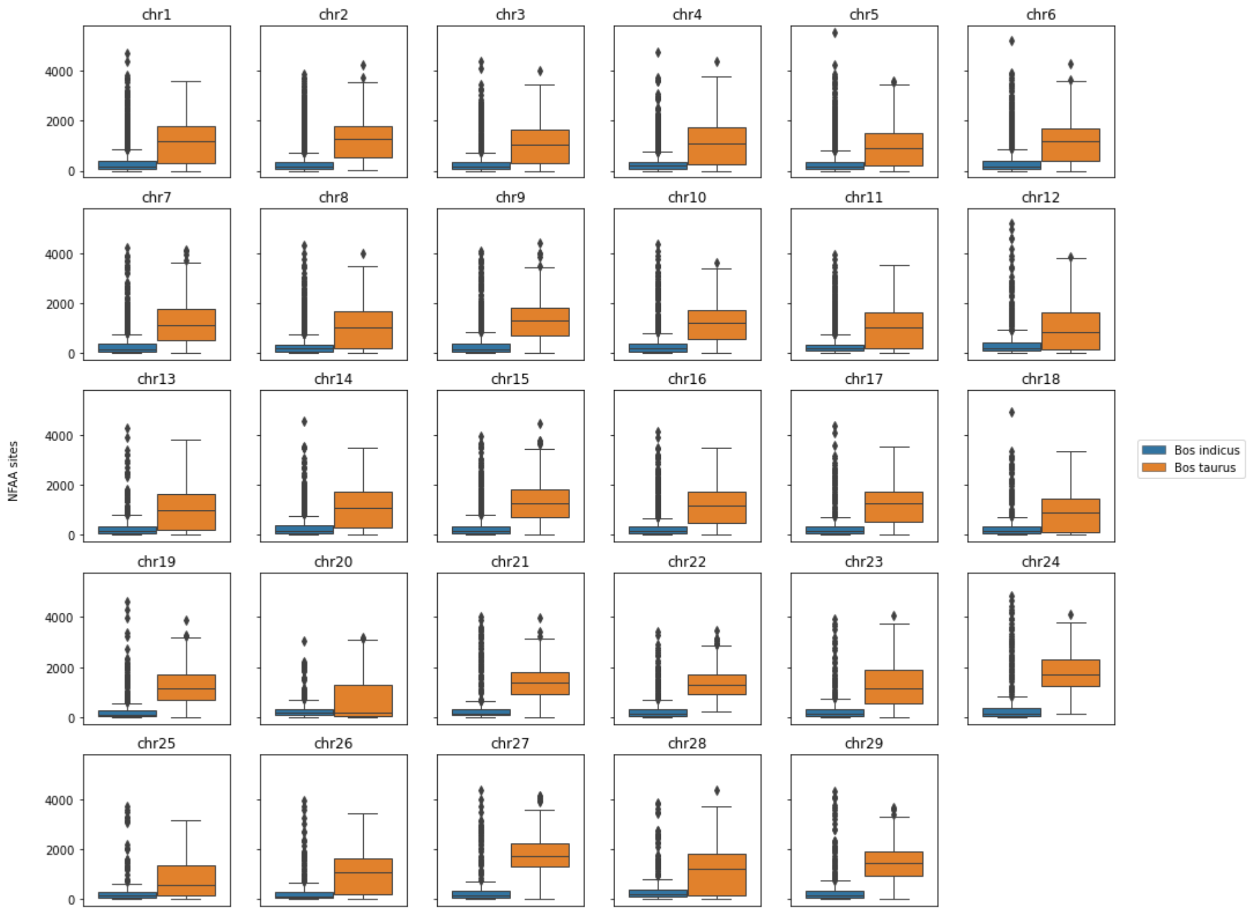

Figure S6. Boxplot of the number of SNV with alternative allele frequency of 0.95 or higher (i.e., nearly fixed alternative allele-NFAA) using UOA_Brahman_1 as the reference sequence (1 Mb scanning windows)

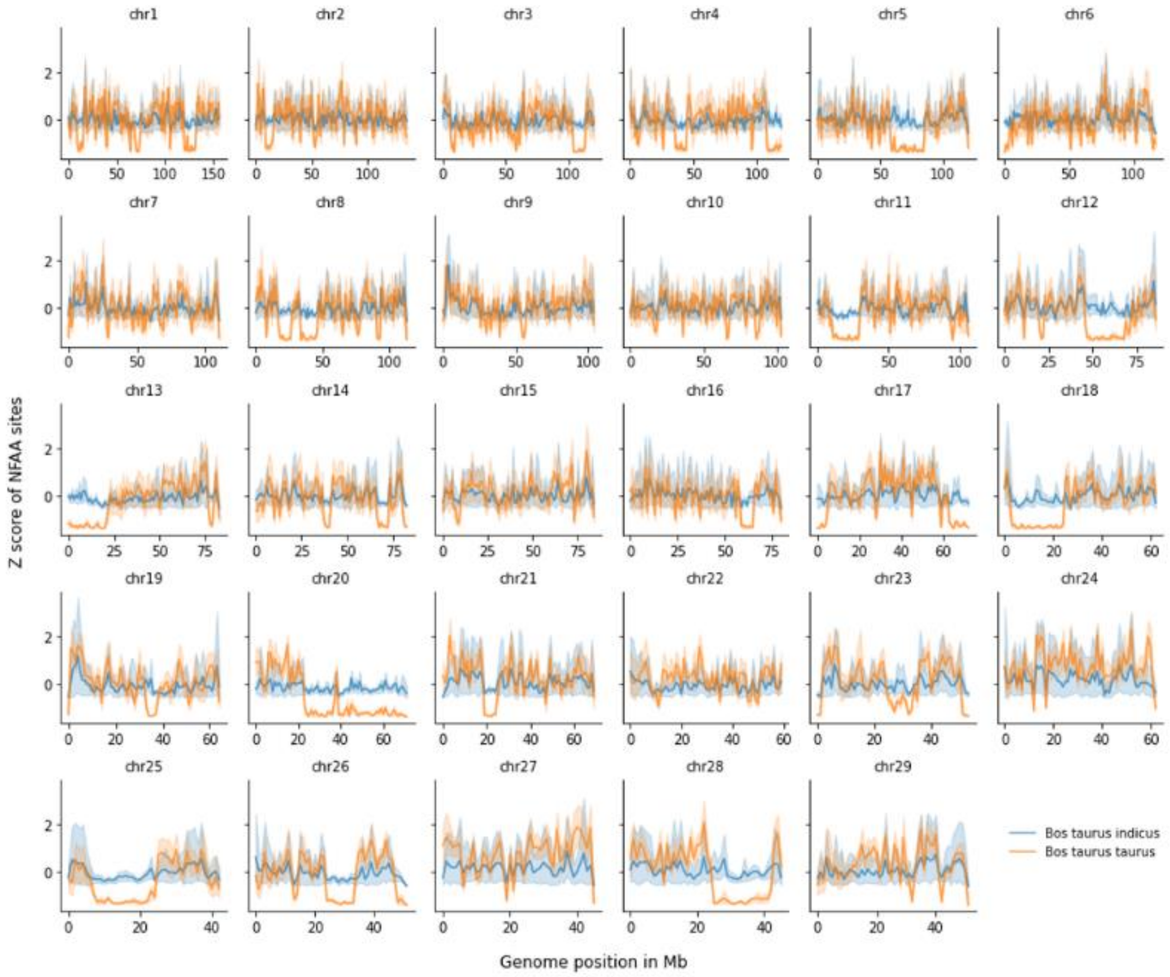

Figure S7. Z-score transformation of the number of SNV with alternative allele frequency of 0.95 or higher (i.e., nearly fixed alternative allele-NFAA) using UOA_Brahman_1 as the reference sequence (1 Mb scanning windows)

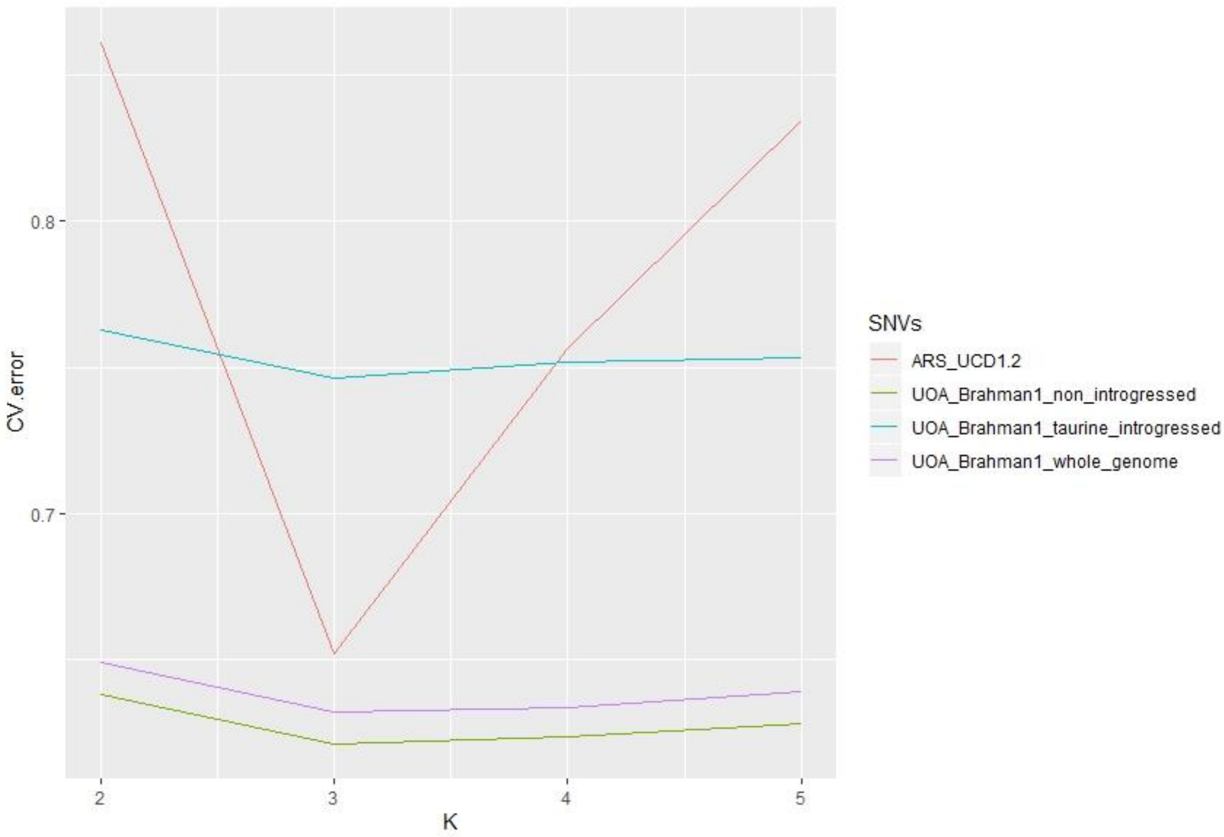

Figure S8. Cross-validation error for admixture analysis using K = 2 to 5 using SNVs derived from whole genome, putative taurine-introgressed, non-introgressed of UOA_Brahman_1, and ARS_UCD1.2
